# Recent innovations in adaptive trial designs: A review of design opportunities in translational research

**DOI:** 10.1017/cts.2023.537

**Published:** 2023-04-28

**Authors:** Alexander M. Kaizer, Hayley M. Belli, Zhongyang Ma, Andrew G. Nicklawsky, Samantha C. Roberts, Jessica Wild, Adane F. Wogu, Mengli Xiao, Roy T. Sabo

**Affiliations:** 1 Department of Biostatistics and Informatics, University of Colorado Anschutz Medical Campus, Aurora, CO, USA; 2 Department of Population Health, New York University Grossman School of Medicine, New York, NY, USA; 3 Department of Pediatrics, University of Colorado Anschutz Medical Campus, Aurora, CO, USA; 4 Department of Biostatistics, Virginia Commonwealth University, Richmond, VA, USA

**Keywords:** Adaptive trials, sample size re-estimation, treatment arm selection, smart designs, master protocols

## Abstract

Clinical trials are constantly evolving in the context of increasingly complex research questions and potentially limited resources. In this review article, we discuss the emergence of “adaptive” clinical trials that allow for the preplanned modification of an ongoing clinical trial based on the accumulating evidence with application across translational research. These modifications may include terminating a trial before completion due to futility or efficacy, re-estimating the needed sample size to ensure adequate power, enriching the target population enrolled in the study, selecting across multiple treatment arms, revising allocation ratios used for randomization, or selecting the most appropriate endpoint. Emerging topics related to borrowing information from historic or supplemental data sources, sequential multiple assignment randomized trials (SMART), master protocol and seamless designs, and phase I dose-finding studies are also presented. Each design element includes a brief overview with an accompanying case study to illustrate the design method in practice. We close with brief discussions relating to the statistical considerations for these contemporary designs.

## Introduction

Clinical trials are considered the gold standard of evidence in clinical research [1,2]. However, modern clinical research problems are becoming increasingly complex while available resources may be limited. Traditional clinical trial designs with fixed sample sizes and only one or two arms may be limited to efficiently address emerging research questions. One such pathway to address these limitations is through the use of “adaptive” clinical trial designs that allow for the prospective modification based on the accumulating data in a trial [1,3–7].

In 2019, the US Food and Drug Administration (FDA) published guidance on the use and implementation of adaptive designs for clinical trials [1]. This guidance included nonbinding recommendations for the design, conduct, and reporting of such clinical trials. The FDA noted that adaptive designs may have multiple advantages over traditional, nonadaptive designs for improving statistical efficiency of trial operating characteristics (e.g., type I error rates and power), addressing ethical considerations (e.g., stopping a trial early if an intervention shows safety concerns or limited benefit, increasing randomization to better-performing arms), adding to our understanding of treatment effects (e.g., enriching a trial with more participants expected to benefit), and being more acceptable to stakeholders (e.g., increased flexibility to successfully complete the trial) [1,8,9]. These adaptive designs have the potential to be applied across clinical trial phases, from phase I dose-finding studies to phase III confirmatory trials [1,2].

This review article first presents the seven major adaptive design elements presented in the 2019 FDA guidance document, with a brief overview of the designs presented in Table 1. A case study is presented in each design subsection to illustrate the approach in practice. We follow this with an introduction of special topics relating to other novel trial designs and methodologies that have emerged regarding borrowing historic information, SMART designs, master protocol and seamless designs, and phase I dose-finding studies. The final section presents general statistical considerations for adaptive trial designs before concluding with a brief discussion.

**Table 1.** Brief summary of FDA guidance document adaptive design elements

Adaptive design element	Brief description	Advantages	Challenges
**Group sequential designs**	Designs that allow for one or more prospectively planned interim analyses of trial data with prespecified criteria for stopping the trial, generally based upon evidence of efficacy/effectiveness or futility	Trial can stop early, reducing overall sample size Results can be disseminated more quickly if trial stops early	May need a much larger maximum sample size than a trial without interim monitoring Early stopping may reduce safety data on a new intervention
**Adapting the sample size**	When uncertainty exists around the estimates used to power a study, an interim analysis can use accumulating data to re-estimate the sample size to ensure a trial has high power if the true magnitude was less than hypothesized but is still clinically meaningful	Reduces chance of a negative trial with a meaningful effect by increasing the sample size to have sufficient power Blinded approaches and re-estimation of nuisance parameters minimally impact type I error rates	Sample size increase may be so large as to be infeasible for continued enrollment Certain approaches may inflate the type I error rate without special consideration
**Adaptive enrichment**	A design which may adapt the patient population to a targeted subgroup (usually through demographic characteristics or by genetic/pathophysiologic markers believed to be related to the mechanism of action) or continue to enroll the participants from the originally specified trial population	Can refine eligibility criteria to enroll subgroups most likely to benefit from the intervention Approaches exist for both prognostic (identifying high-risk patients) and predictive (identifying more responsive patients) enrichment	Subgroups may be small (i.e., rare or hard to enroll) and challenging to determine benefit Choice of demographic characteristics or genetic markers may lead to different subgroups
**Adaptations to treatment arm selection**	Modification to the trial design that could add or terminate study arms, present in both early phase studies (e.g., dose-finding) and later phase studies (e.g., seamless designs and platform trials)	Extremely flexible to terminate study arms (for futility or efficacy) and add new arms Shared control arm, if used, may increase allocation to study interventions Can use a single master protocol versus multiple standalone trials	Multiple comparisons may lead to issues with type I error rates Criteria for terminating arms or adding new arms may be complex Overall resource use may be challenging to plan for given unknown number of arms and sample size
**Adapting patient allocation**	Also known as adaptive randomization (AR), the incorporation of methods to modify the randomization process that may be based on baseline covariates (i.e., to achieve “balance” in select covariates across study arms), response/outcome AR (i.e., attempting to randomize more participants to “effective” arms), or maintaining equal amounts of information when incorporating historic/supplemental data sources	Covariate-AR only uses baseline characteristics to promote balance across groups Response-AR increases probability of allocation to better-performing study arms AR when incorporating external control arm data can increase allocation to the intervention to maintain a desired allocation (e.g., 1:1)	Need to account for Covariate-AR in the statistical analysis plan Response-AR has special challenges with temporal trends, two-arm studies, & potential unblinding of study arms To incorporate external data advanced statistical approaches are needed and this supplemental data may introduce bias
**Adapting endpoint selection**	The ability to select one endpoint from a collection of potential primary endpoints when there is uncertainty about effect sizes across outcomes at an interim analysis, when done in FDA trials it involves extensive discussion and the review with the FDA Review Division	Can select a primary endpoint while the trial is ongoing based on uncertainty in the design stage Recent statistical research explores new designs to incorporate this adaptation	Not recommended by the EMA FDA recommends review and discussion with regulatory authorities May be seen as cherry picking an outcome that makes the intervention look good
**Adapting multiple features**	The above elements can be utilized individually or may be combined within a single adaptive trial design (at the expense of increasing complexity that needs to be carefully and thoroughly evaluated)	Provides the greatest flexibility by combining multiple adaptive features May result in designs with greater appeal to participants	Combining multiple adaptive features requires extensive statistical work and simulation studies May result in overly complex studies when a traditional design may have sufficed

## FDA Adaptive Trial Elements

### Group Sequential Designs

Group sequential designs allow for prospectively planned interim analyses and possible early stopping due to efficacy, futility, or harm [1]. These stopping rules may be binding (e.g., the trial must stop) or nonbinding (e.g., the trial is recommended to stop, but other considerations may warrant continuing the study). Since the early works of Pocock [10] in 1977 and O’Brien and Fleming [11] in 1979, the choice of efficacy-stopping boundaries that control the overall type I error rate have been broadly discussed in the literature (e.g., Lan and DeMets [12,13]; Wang and Tsiatis [14]; Jennison and Turnbull [15]; Chow and Chang [5]). With regard to futility stopping rules, either binding or nonbinding, type I error rate control is guaranteed but type II error may be inflated [16].

The related statistical approaches can be divided into frequentist methods, including conditional power [17] and beta-spending functions [18–21]; and Bayesian methods based on the predictive power [22], posterior, or predictive probability [23]. Variations of adaptive group sequential designs have been developed with modifications of sample size [24–26], treatment arm selection [27–29], and patient enrichment [30,31] and are discussed in later sections. It is worth noting that conventional methods tend to produce biased estimates and undesirable coverage probability of confidence intervals; therefore, various methods for the estimation of the treatment effect that appropriately adjust for group sequential stopping rules have been established [1,15,32].

***Case Study:*** PARAMEDIC2 was a Phase III randomized, placebo-controlled trial to test the efficacy of epinephrine in out-of-hospital cardiac arrest patients on 30-day survival rates (EudraCT number 2014-000792-11) [32,33]. A frequentist group sequential design was used with a total of 10 pre-specified interim analyses spaced every 3 months. Since epinephrine was the standard treatment, a higher level of evidence was required to stop early for futility. To account for this different expectation of evidence for futility and efficacy, asymmetric stopping boundaries were implemented including Pocock’s and O’Brien Fleming’s alpha-spending functions for efficacy and futility, respectively [32]. Although epinephrine showed a significantly higher survival rate in the final analysis, the trial missed an opportunity to stop early for efficacy due to slower recruitment and lower survival rates than were expected when designing the study [33,34]. Through simulation studies, the trial was re-constructed using three alternative Bayesian group sequential designs where the stopping decisions were based on the posterior probability of superiority and harm [34]. The team then virtually re-executed the trial using PARAMEDIC2 data where only two designs recommended stopping early for benefit.

### Sample Size Re-Estimation

Sample size re-estimation (SSR) is a form of clinical trial adaptation that accounts for potential uncertainty in the expected treatment effect. After the initial sample size calculation is performed to estimate the number of participants required to maintain prespecified power and type I error rate given an assumed treatment effect, the accumulated data at each interim analysis can then be used to re-estimate the sample size in order to maintain the same power if the observed treatment effect is weaker than expected but still clinically relevant. Many distinct methods exist to perform SSR, including blinded (i.e., individual group assignment remains hidden in the process of re-estimating) and unblinded (i.e., assignment is known for the process of re-estimating) methods [35]. Bayesian SSR methods also exist [36,37], as do methods extending SSR to complex study designs [38–40], less common statistical models [41], and time to event data [42]. SSR is most commonly used in mid to large scale phase II/III confirmatory trials [43]. Further, re-estimation methods can be tailored to equivalence, noninferiority, or superiority hypothesis testing contexts [44–46]. Because it relies on interim analyses from incomplete data, SSR may inflate the type I error rate [47]. SSR which depends on re-estimating only nuisance parameters (e.g., variance) or uses a blinded approach has little impact on type I error rates, but unblinded methods may lead to an inflation of the type I error rate [48–50]. This type I error inflation can be addressed differently depending on how the re-estimation is performed and is always an important consideration when planning or implementing SSR. Used appropriately, SSR allows for uncertainty in the magnitude of the treatment effect, letting trials readjust their total sample size throughout the trial based on accumulated information without loss of statistical power.

***Case Study:*** One adaptive trial with a planned SSR element is the ongoing Nasal Oxygen Therapy After Cardiac Surgery (NOTACS) trial (ClinicalTrials.gov number: NCT05308719) [51]. This trial is evaluating the use of high-flow nasal oxygen therapy (HFNT) compared to standard oxygen therapy (SOT) for cardiac surgery patients at increased risk for postoperative pulmonary complications. The sample size for this two-armed trial is restricted to be between 850 and 1252 patients, with a single interim sample size re-estimation analysis planned after at least 300 patients have accumulated 90 days of follow-up. This design allows the trial to increase the sample size above 850 patients if, based on interim data, the treatment effect of HFNT is lower than originally expected but still clinically meaningful enough to justify continuing the trial with the necessary higher sample size to maintain statistical power to detect an effect.

### Adaptive Enrichment

In trials where it is believed that a subgroup of the population will experience more benefit from an intervention, an adaptive enrichment design provides the ability to drop lower performing subgroups at an interim analysis so that study resources are more efficiently allocated to those with a greater chance of benefit [1,9,52–54]. In general, two broad classes exist: prognostic and predictive enrichment [9]. Prognostic designs attempt to choose patients more likely to have the study endpoint or have worsening conditions (e.g., event- or progression-based studies), whereas predictive designs attempt to identify patients with a higher chance of responding to the given treatment [9].

These designs are most common for confirmatory clinical trials, but may be used in earlier phases [1,54,55]. The *a priori* subgroups may be identified by either a single biomarker or multiple biomarkers, but designs may also include methods to identify adaptive thresholds for continuous biomarkers [52,53]. An advantage of enrichment designs compared to a study that only enrolls the targeted subgroup is that the overall population, including nontargeted subgroups, may be evaluated for potential benefit while providing the ability to drop the nontargeted subgroups with no benefits or worsening conditions [1]. A challenge of enrichment designs is accounting for the multiplicity of both repeated testing of study hypotheses and across multiple subgroups [1,52,54]. Burnett and Jennison discuss approaches to control the type I error rate within an adaptive enrichment trial, including a strong family-wise control [54]. Regardless of whether study arms are dropped at an interim analysis, the data from the overall study may be used for statistical inference [1].

***Case Study:*** An example of a clinical trial that included an adaptive enrichment element is the Phase III Trial of TRC105 and Pazopanib Versus Pazopanib Alone in Patients With Advanced Angiosarcoma (TAPPAS; ClinicalTrials.gov number: NCT02979899) [56]. Due to uncertainty of the treatment effect for progression-free survival (PFS) among cutaneous and noncutaneous disease subgroups of angiosarcoma, an adaptive enrichment strategy based on conditional power was used after 40 events had occurred or within 30 days after enrollment of 120 participants to determine if the trial should continue without any modifications, should potentially increase the overall sample size (i.e., sample size re-estimation), or if the noncutaneous subgroup should be dropped with an increase of the sample size for the cutaneous group [56]. While there was no formal criteria for stopping for futility, the TAPPAS Trial design specified an “informal futility zone” that the Data Monitoring Committee for the study could use to stop the trial at an interim analysis [57]. Ultimately, the trial terminated for futility after 123 participants were enrolled because the study had entered the informal futility zone with a hazard ratio of 0.98 for PFS between the two groups (*p* = 0.95) [56].

### Treatment Arm Selection

Adaptations to treatment arm selection are methods that modify the study design to allow for adding or dropping treatment arms. Some examples are adaptive dose-finding, drop-the-losers, adaptive seamless, and adaptive platform designs [4,6]. These adaptations can be used in both Bayesian and frequentist trial designs. Some of the applications are described below, as well as in subsequent sections detailing additional considerations of these types of design modifications.

Adaptive dose-finding designs are usually used in the early phases of studies to determine the minimum effective dose or the maximum tolerable dose for a drug to use for future trials. One method used for dose-finding designs is the continual reassessment method where the dose relationship is assessed throughout the trial using the data collected during the study [58].

The adaptive seamless design combines the objectives of two trials into one. Seamless design trials can combine phases I and II or phases II and III. This type of design eliminates the time between the two phases of a trial and can lead to a smaller overall sample size required to conduct the final analysis where it uses data collected from subjects enrolled before and after the adaptation [59].

Drop-the-losers clinical trial designs allow the dropping of the inferior treatment(s) or adding additional treatment arms. This is usually a two-stage trial and during the first stage of the trial, the inferior treatments are dropped based on pre-specified criteria or by conducting interim analysis. The winning treatment is then tested against a control [60].

Adaptive platform trial designs study multiple interventions for a common targeted condition. Based on a decision algorithm, treatments can be removed or added to the trial. The number and type of treatments can change throughout the study period. The benefit of this design is that the focus is on the condition rather than the treatment [61,62].

***Case Study:*** Cocaine use disorder (CUD) is a difficult condition to treat, and research has not found an effective treatment for the condition. Suchting *et al*. designed a drop-the-loser (DTL) Bayesian adaptive trial to determine the most effective dose of citalopram, a selective serotonin reuptake inhibitor that can treat CUD, to evaluate in a larger confirmatory trial (ClinicalTrials.gov number: NCT01535573) [63]. Using Bayesian posterior probabilities, the researchers decided at the interim analysis which doses would be dropped from the trial (20 mg/day; 40 mg/day). In the implementation of the trial, the 20 mg/day dose was dropped after 50% of recruitment given that the 40 mg/day dose had a higher chance of success. Ultimately, the 40 mg/day dose provided “moderate-to-strong evidence” of positive effects at study conclusion [63].

### Adaptive Randomization

Randomization is the foundational basis for establishing efficacy of an investigational treatment in a randomized controlled trial (RCT). In theory, the randomness element seeks to establish study arms with similar patient characteristics between arms such that the only differing factor is the treatment assignment. Critical to the success of randomization is ensuring appropriate sample size allocation across arms as well as adjusting for any baseline covariates for which it is important that there is no imbalance.

Several randomization approaches in RCTs exist: The simplest approach is the coin flip; however, this method fails to control allocations across study arms and does not offer any direct covariate adjustment. To correct these issues, the idea of stratified block randomization emerged in the mid-twentieth century [64,65]. This approach uses pre-specified randomization sequences within subgroups of patients and is relatively straightforward to implement. However, some limitations to stratified block randomization remain, namely requiring continuous stratification variables to be categorized, an inability to accommodate a large number of stratification covariates, and increased risk of selection bias near the end of a block when the allocation may be predicted [66].

To address the limitations of stratified block randomization, covariate-adaptive designs were created to marginally balance multiple covariates at once rather than achieve balance within each stratum [67]. The minimization method is a covariate-adaptive method which was originally proposed by Taves [68] and Pocock and Simon [69]. It implements an approach to minimize imbalance for each covariate of interest, thus allowing for more flexibility and options including weighting of covariates [70,71]. However, minimization has been criticized for being more challenging to implement than stratified block randomization, requiring investment in software and personnel time from a study statistician.

Another strategy for randomization is the response-adaptive design, which modifies the allocation ratios based on the accumulating evidence of success in the trial arms [72,73]. This does not ensure balance of the baseline covariates, but attempts to allocate more participants to potentially effective study arms. Korn and Freidlin note adaptive randomization methods have the potential for increasing the total number of nonresponders relative to equal fixed allocation designs (i.e., 1:1 allocation). Further, response-adaptive randomization may introduce bias from temporal trends, lead to the unblinding of study arms and patients assigned to inferior arms due to variability in estimating effects based on smaller sample sizes [74,75]. Recent work noted that response-adaptive randomization may be less susceptible to these issues in multiarm trials where the control maintains a fixed allocation, but caution should still be taken in implementing these methods [76].

***Case Study:*** The Established Status Epilepticus Treatment Trial (ESETT; ClinicalTrials.gov number: NCT01960075) randomized participants to three intravenous anticonvulsive agents with an initial 1:1:1 allocation that changed based on a response-adaptive randomization design after 300 participants were observed [77]. ESETT also allowed for early termination for success or futility based on predefined stopping rules. After 300 participants, the randomization ratios were updated based on accumulating data to increase allocations to what appeared to be more promising study arms. However, the study was terminated for futility after 384 unique participants and it was determined that the probability of meeting full enrollment and detecting a significant effect was minimal [77].

### Endpoint Selection

Endpoint selection is a critical part of designing a clinical trial. In adaptive clinical trials, in addition to the trial’s feasibility, its cost, and the intended goal of treatment, the modifications of trial procedures or statistical methodologies will impact the selection of endpoints and outcomes, especially for adaptive designs of confirmatory clinical trials [7,78].

Whether it is selected at the beginning of the trial or adapted during an on-going trial based on comparative interim results, the primary outcome selected for evaluation, (1) must address the trial objective and should be acknowledged as meaningful to clinicians, patients, and policymakers and (2) must be supported by enough scientific evidence to demonstrate its clinical relevance, i.e., it represents a current and reliable measure of clinical benefit in the target population [1,79].

Adaptation of the endpoint selection might be motivated by the uncertainty about the treatment effect sizes on multiple patient outcomes that are all considered acceptable as primary endpoints for a trial [1]. Regulatory and institutional guidelines elucidate directions in implementing the adaptation of endpoint selection, such as the adaptation rule should be pre-specified, and statistical hypothesis testing should account for the adaptive endpoint selection, as stated in the FDA’s 2019 Adaptive Trials Guidance [1]. Additionally, the FDA notes early discussion with the FDA review division is encouraged before considering adaptive endpoint selection methods [1]. On the other hand, the European Medicines Agency’s (EMA) Committee for Medicinal Products for Human Use (CHMP) in general warns against changing the primary endpoint in adaptive trials, stating the difficulty to justify changing the primary endpoints as they are chosen to describe a clinically relevant treatment effect and/or clinical benefit, which are defined during the study’s planning stages and cannot be changed based on interim results of the study [80].

***Case Study:*** The use of adaptive endpoint selection is challenging with no straight-forward examples that the authors are aware of currently published in the literature. This may be due to the recommendation to consult with regulatory authorities, such as the FDA, prior to initiating such approaches or the strong recommendations against outcome adaptations from the EMA to avoid the appearance of bias or cherry picking by study investigators.

However, work is being done to move towards designs and contexts where adaptive endpoint selection may be advantageous. Filozof *et al*. discuss the scientific and logistical usage of an adaptive design-based approach to develop therapeutic strategies for patients with nonalcoholic steatohepatitis (NASH) [81]. The development of drugs for NASH has been substantially slow for a number of reasons, such as the heterogeneous nature of NASH with respect to the risk of progression to cirrhosis and the lack of a validated surrogate endpoint to clinical outcomes. Filozof *et al*. argue that, given the high unmet medical need and the lack of validated surrogate endpoints in NASH, the use of adaptive endpoint selection design methods appears reasonable as they provide the flexibility and efficiency for identifying potential signals of clinical benefit of the test treatment [81].

Recent work has been done to evaluate the statistical properties and potential benefits of these designs in certain contexts. Xu *et al*. proposed a design for rare diseases that maintains the family-wise type I error rate when selecting a primary endpoint based on an internal informational cohort when limited prior data exists and separate natural history cohort studies may be expensive or challenging to conduct [82]. Roig *et al*. proposed an adaptive design that allows the modification of the primary endpoint based on blinded interim data while also recalculating the sample size accordingly [83].

### Adaptive Multiple Features

A clinical trial may also include multiple adaptive design features. As with any single trial design feature, it is important to ensure that the trial operating characteristics maintain the desired type I error rate and statistical power while enrolling a realistic sample size across a range of plausible future scenarios. Often, these are evaluated through extensive simulation studies [1].

***Case Study:*** The vitamin C, thiamine, and steroids in sepsis (VICTAS; ClinicalTrials.gov number: NCT03509350) randomized clinical trial enrolled adults with sepsis-induced respiratory or cardiovascular dysfunction to examine if treatment with vitamin C, thiamine, and hydrocortisone result in more ventilator- and vasopressor-free days relative to placebo [84,85]. The design incorporated both an adaptive sample size selection and Bayesian predictive probability monitoring for futility or efficacy [85]. If fewer than 400 participants were enrolled, interim monitoring would only consider stopping for efficacy, whereas sample sizes above 400 would consider stopping for either futility or efficacy [84]. Ultimately, the trial terminated after 501 participants due to a change in funder’s priorities and no further enrollment occurred and the criteria for statistical significance not being met [84]. This represents an unexpected conclusion to the study, but illustrates the potential for multiple adaptive elements to be included in one study.

## Other Novel Trial Designs and Adaptations

### Incorporating External/Supplemental Information

Information or data external to a trial may be useful to incorporate into analyses of a trial in order to increase the effective sample size. Some designs may attempt to incorporate this information to increase the sample size of treatment arms in the study, generally based on evaluating the exchangeability (i.e., equivalence) of the supplemental data with the current study data. Many approaches exist and include multisource exchangeability modeling [86,87], commensurate priors [88], power priors [89,90], and general Bayesian hierarchical models [91]. It is also possible to include adaptive randomization methods when information is borrowed to maintain the overall proportion of trial data in a predetermined ratio (e.g., if historic control data are borrowed, more participants could be randomized to treatment arms) [92]. When considering methods that incorporate supplemental information, one must consider the sensitivity of methods to downweight nonexchangeable sources as to avoid potentially biasing the results towards historical data [86]. Additional challenges may be the specification of priors in study protocols given the uncertainty of future scenarios, but methods exist to account for this uncertainty in the design stage and to calibrate hyperparameters or tuning parameters [93].

***Case Study:*** Putative Investigational Therapeutics in the Treatment of Patients With Known Ebola Infection (PREVAIL II; ClinicalTrials.gov number: NCT02363322) was a platform trial designed to sequentially evaluate potential therapies for the treatment of Ebola virus disease in the context of the West Africa Ebola outbreak in the mid-2010s with Bayesian posterior probabilities to facilitate frequent interim monitoring [94]. Given the sequential nature of the design, each “new” platform would always have at least one “old” platform of data that was just completed, but these data were not used in the PREVAIL II design due to concerns about changing disease outcomes over time. Kaizer, Hobbs, and Koopmeiners proposed a modified design that allowed for incorporating information from past platforms while also adapting the randomization ratio to enroll more participants to the new intervention if historic data were borrowed for the current control [92]. They demonstrated that information sharing could more quickly identify potentially effective therapies while randomizing more participants to the new arms with minimal bias based on the multisource exchangeability models used [92].

### SMART Designs

The sequential multiple assignment randomized trial (SMART) is an adaptive research design for building optimal adaptive interventions. The general framework of a SMART design is as follows: every participant is randomized to an intervention arm initially, similar to the start of a classic, fixed RCT. Following this initial assignment, patients move through a series of stages with the option to either stay on or switch intervention arms, depending on their response to the intervention in the stage prior. If a participant switches intervention arms at the next stage, they will be re-randomized to a new treatment to maintain properties of causal inference associated with randomization within an RCT. The SMART framework mimics standard clinical practice in that, with time, patients will be assigned to more effective treatments. The SMART design therefore not only helps to build optimal dynamic treatment regimens, but also permits researchers to identify characteristics of patients for whom particular treatment regimens may be most effective [95,96]. SMART designs, while not technically adaptive since there are no prospectively defined modifications to the *design*, do represent an emerging class of designs that address pressing clinical questions relating to precision medicine and identifying the optimal treatment for any given patient.

***Case Study:*** SMART designs are traditionally used in fields such as precision and behavioral medicine, mental health, or substance use where different combinations and sequences of treatments are administered [97]. The Establishing Moderators and Biosignatures of Antidepressant Response in Clinical Care (EMBARC; ClinicalTrials.gov number: NCT01407094) study is one example of a study that employed a SMART design [98]. The primary aim of this two-stage, multisite, 8-week duration study was to discover candidate biomarkers as moderators of antidepressant treatment among depressed patients. A SMART design was selected due to the goal of investigating possible combinations of biomarkers and clinical characteristics as mediators and moderators to generate biosignatures for making personalized medication treatment prescriptions [99]. Meanwhile, data were collected 1 week after randomization to provide early indicators of patient response to treatment and thus to refine any subsequent treatment adaptations and predictions regarding the treatment response.

### Master Protocol Designs

Master protocols are single, comprehensive trials developed to evaluate several concurrent sub-trials, involving assessment of multiple therapies, diseases, or subpopulations [100–104]. There are three general types: basket, umbrella, and platform trials. Basket trials evaluate single therapies across multiple diseases with common molecular alterations [100,105]. Sub-studies are often similarly designed single-arm trials, allowing pooled safety and efficacy data across subpopulations [101,104]. Umbrella trials evaluate multiple therapies for a single disease stratified into subgroups [100,102,106]. Substudy designs can be single- or multiarm, though often include a control [104,107]. Platform trials investigate multiple therapies in one or more diseases in an ongoing manner with arms added or dropped as new data and evidence appear [61,101,106], often using Bayesian methods based on probabilities of treatment success or failure [61,108]. These designs may all include adaptive elements (e.g., arm dropping or group sequential methods) or be nonadaptive in their implementation.

Master protocols contain common guidelines for enrollment, measurements, data management, and statistical analysis; create a shared infrastructure across treatments; can reduce costs and increase efficiencies; and allow recruiting broader patient populations, helping bridge the translational gap toward clinical care [61,99,101,104,109]. Challenges include controlling false discovery rates [106,108,110,111], population drift [61,101,108], coordination among multiple partners [109], intensive review and monitoring processes [103], amendments [112], and informed consent [103,112].

***Case Studies:*** NCI-MATCH (Molecular Analysis for Therapy Choice; ClinicalTrials.gov number: NCT02465060) is a phase II basket trial for patients with advanced refractory solid tumors, lymphomas, or multiple myeloma [113]. The Lung Cancer Master Protocol (Lung-MAP; ClinicalTrials.gov number: NCT02154490) is an umbrella trial for patients with advanced squamous nonsmall cell lung cancer, consisting of two-arm, seamless phase II–III randomized substudy designs [114]. I-SPY-2 (Investigation of serial Studies to Predict Your Therapeutic Response with Imaging and Molecular Analysis 2; ClinicalTrials.gov number: NCT01042379) is a multicenter phase II platform trial for patients with local metastatic breast cancer breast cancer, with promising drugs graduating to Phase III using Bayesian predictive probabilities [115–117].

### Seamless Designs

Therapeutic development often occurs in distinct phases, with pauses between the “learning” and “confirming” phases [3,118–120]. Seamless designs combine phases into a single protocol with no pause [3,121]. Operationally seamless designs combine phases into one trial, while inferentially seamless designs also combine data from distinct phases [3,118]. Seamless phase I/II designs simultaneously assess toxicity and efficacy [122], while seamless phase II/III designs combine the exploratory and confirmatory stages [123], where futile treatments are dropped and promising treatments are investigated further [3,118,120].

Seamless designs can accelerate the development timeline, reduce total sample size, and better estimate the risk of adverse events [3,118–120,124]. These designs require planning across stages, including prespecification of protocols, interim analysis schedule, statistical analysis plans, and data safety monitoring [3,119], which can constrain flexibility and adaptability [124]. Poor selection of short-term surrogate outcomes in early stages adversely affects later stages [124,125]. Statistical methods that control type I error rates are required to reduce false positives [3,120]. Seamless designs are generally appropriate for nonpivotal trials or for therapies with sufficient preliminary evidence [119,124].

***Case Studies:*** The Seamless Phase I/II Randomized Design for Immunotherapy Trials (SPIRIT) was proposed to investigate an immunotherapeutic agent that acts against the programed cell death ligand 1 checkpoint inhibitor in recurrent ovarian cancer [126]. The first stage determined the range of safe doses using a Bayesian optimal interval design, while admissible doses were explored in the second stage jointly modeling immune response and PFS [127]. A seamless Phase IIb/III trial for chronic obstructive pulmonary disease identified treatments that exceeded a threshold for clinical relevance and were superior to control based on forced expiratory volume, then provided further evaluation based on percentage of days with poor control [128].

### Phase I Studies

Clinical trials are defined by a series of phases depending on their overarching goal. A phase I clinical trial is a preliminary stage in this sequence characterized by its purpose to establish safety of an intervention and dosage in the case of a new drug. This includes identifying the maximum tolerated dose (MTD) which may be carried forward for future studies. Typically, these trials enroll a small number of subjects, thus utilizing designs that maximize the number of resources available is paramount.

Adaptive designs such as model-based or model-assisted approaches incorporate prior knowledge using Bayesian principles to allow for efficient use of data collected during the trial [129]. One such dose-finding design is the Bayesian Optimal Interval (BOIN) and its extensions [127]. The BOIN design targets pre-specified toxicity level and with optimal boundaries. Based on the observed toxicities exhibited in a dosing cohort, the BOIN design recommends the optimal dose level for the next sequence of patients. Model-assisted designs generally provide an easier to interpret framework to clinicians as all operating characteristics can be pre-tabulated [129]. However, model-based approaches can require more input during the conduct of the trial as they need repeated model fitting and estimation. Care must be taken in the assumptions made to implement the design with clinical input and good statistical practice informing the final product, such as the type I error rate control. Design choices must be made *a priori* to adhere to the operating characteristics of the design.

Adaptive Phase I designs yield improved accuracy for identifying the MTD relative to standard approaches such as the 3 + 3 and allocate more patients to correct dosage level, improve safety outcomes, and increase the amount of information available for subsequent trials [129]. Recent advancements have also explored the use of “expansion cohorts” which take the identified dose from phase I seamlessly into an expanded phase II clinical trial [130].

***Case Study:*** A phase I trial examined the optimal dose of TG02 and temozolomide in treating high-grade gliomas that are traditionally highly resistant to treatment (ClinicalTrials.gov number: NCT02942264) [131]. A BOIN design was used with a target dose-limiting toxicity rate of 35% for the MTD. Ultimately, 38 participants were included and the combined dose for the two treatments was identified to use for designing a future phase II randomized trial for evaluating efficacy [131].

## Statistical Considerations

### Statistical Analysis for Adaptive Designs

Analysis for adaptive trials generally involves computing a suitable treatment effect estimator, inferences about the estimated effect, and the type I error rate control [132]. The typical maximum likelihood estimation (MLE) for the treatment effect in the fixed sample size trial may be biased in adaptive designs because of the trial design adaptation (e.g., stopping rule) and selection of promising subgroups following interim analyses [133]. Robertson *et al*. provided an overview of treatment effect estimators that improve the bias over MLE and their use in various types of adaptive designs [133]. Statistical inference of the point estimate needs to have a correct coverage of confidence intervals (CIs) and account for design realizations such as multiple stages [15,134–136]. Similar to CIs, hypothesis tests and p-values are combined across multiple stages of adaptive designs based on the conditional invariance assumption and it is advisable to pre-specify methods to generate p-values in trial protocols [15,132]. During hypothesis testing, the type I error rate needs to be adjusted when testing multiple hypotheses or when choosing a subpopulation or treatment, especially among confirmatory clinical trials [1,137–139].

The above discussion so far focuses on the frequentist framework. However, Bayesian inference is commonly employed in adaptive designs since it can maintain the desired trial operating characteristics as demonstrated through simulation studies and has an adaptive nature [1,140,141]. For analysis implementation, software solutions in adaptive designs are summarized by Danielson *et al*. in Chapter 12 [142]. In practice, the adaptive nature of a design should be accounted for in the analysis plan for the trial to avoid potential bias and unexpected coverage levels.

### Trial Planning and Sample Size Calculations

A common theme throughout many of the designs is the importance of *a priori* planned adaptations and the identification of the target sample size. Adaptive trial designs need to account for the potential impact on the trial operating characteristics of making mid-trial changes [1]. This is most efficiently done via statistical simulation studies, but more traditional power calculations that do not account for the adaptations may serve as a convenient starting point to explore what changes (e.g., increased sample size) may be needed [1,3]. As with any clinical trial, a range of possible scenarios should be explored with the resulting power and type I error rates summarized to determine if the properties are acceptable for a given context. These findings should be included in the study protocol with all assumptions and design elements clearly identified so that the findings are reproducible.

### Secondary Endpoints

Secondary endpoints are often tested using either gatekeeping or hierarchical testing strategies in conventional trials as interim analysis or trial extension is not intended [143–145]. These approaches are pre-specified in the study protocol, hence the overall type I error rate is strongly controlled. In adaptive trials, these techniques may not maintain a strong control of the overall type I error rate [136]. Hung *et al*. present a procedure for the statistical inference to test secondary endpoints after the primary endpoint reaches statistical significance, which is more complex in adaptive trials [145]. This complexity often arises from the fact that most secondary endpoints are correlated with the primary endpoint and hence more sophisticated approaches to adjust secondary endpoint analyses should be implemented [137].

Appropriate caution should be taken along with the follow-up of proper regulatory guidelines in the analyses, adjustments, or interpretations of secondary endpoints. Further, if the adaptive trial requires the revision or modification of an endpoint, be it primary or secondary, the decision to revise or modify the endpoint should be independent of the data obtained from the trial and should not put the validity and integrity of the trial in question [146,147].

### Safety Considerations

Adaptive design elements may affect the availability of safety information for each study arm (e.g., terminating early for efficacy may not provide sufficient information to evaluate risk vs. benefit) or the adaptive element may place participants at excessive risk (e.g., early phase dose-escalation studies that permit rapid escalation) [1,136]. Where appropriate, a data safety monitoring board should be used to provide an external source of guidance and recommendation as to the safety of the overall study and to make recommendations to the trial investigators for potential modifications. As with any trial, the safety of participants is of utmost importance and adaptive designs should carefully consider trial modifications in the context of potential tradeoffs with participant safety.

## Discussion

There is great potential for adaptive trial designs to improve the efficiency of clinical trials for future research problems with increasing complexity while potentially using fewer resources. Given the wide range of potential adaptations and emerging trial designs, it is clear that numerous designs for trials could be proposed based on the combinations of different adaptive elements. In general, while adaptive trials may provide increased flexibility, there is a need for sustained statistical support to ensure any prospectively planned modifications are made appropriately based on the accumulating data. Table 2 further highlights some of the general advantages and challenges in the implementation of adaptive trial designs. It is also worth noting that not all research questions warrant an adaptive design, and that nonadaptive designs may be more appropriate.

**Table 2.** General advantages and challenges of adaptive trials

Advantages	Challenges
Improved flexibility More efficient use or allocation of available resources (e.g., financial or administrative) Improved statistical efficiency that can provide greater statistical power to detect a true drug effect Ethical considerations may be more readily addressed Ability to answer broader questions, that may be refined as the trial progresses, relative to nonadaptive designs Stakeholders may be more willing to support studies with adaptive elements because of the added flexibility	Advanced and specific analytical methods need to be used to avoid type I error rate inflation (i.e., identifying an ineffective intervention as effective) and control bias in estimates Gains in efficiency generally represent a tradeoff with other trial components (e.g., interim analyses may decrease *expected* sample size at the expense of an increase to the *maximum* sample size) Logistics to ensure appropriate trial conduct and integrity Adaptation may be limited by scientific or clinical constraints or make interpretation more challenging

Adaptive designs have the potential for furthering patient-centered research and recruiting more generalizable study populations. Modifications such as early termination or sample size re-estimation that aim to avoid wasted resources, either by stopping early due to substantial evidence or increasing the sample size to detect clinically meaningful differences, allow research results to more quickly disseminate to the communities which stand to benefit most. Designs with enrichment, adaptive randomization, or treatment arm selection attempt to address ethical concerns with randomizing individuals to arms or enrolling subgroups which may not benefit from the treatment, but special statistical considerations need to be made to maintain the trial operating characteristics. Currently, many adaptive designs examples occur in preclinical and clinical research (translational phases T1 and T2), but the adaptive methods may also be beneficial for clinical implementation and public health research (translational phases T3 and T4) to improve their efficiency, increase representation among diverse groups, and provide increased flexibility.

In summary, we provided a brief introduction to various adaptive design elements and emerging novel trial approaches with accompanying case studies to provide examples of the designs in practice. Additional novel designs and concepts are constantly emerging in the face of new challenges to address various research questions, and this article may serve as a starting point to introduce some of the design considerations to be used in practice. Finally, when designing any clinical trial, care should be taken to ensure the safety and integrity of the study for participants and the statistical trial operating characteristics.
